# Supplementary material for: Overexpression of β-Ketoacyl CoA Synthase 2B.1 from Chenopodium quinoa Promotes Suberin Monomers’ Production and Salt Tolerance in Arabidopsis thaliana
Source: Int J Mol Sci. 2022 Oct 30;23(21):13204. doi: 10.3390/ijms232113204 (PMC9659239; doi:10.3390/ijms232113204)
Supplement: Supplementary file 1 [file ijms-23-13204-s001.zip › ijms-1936602-supplementary.pdf]

International Journal of *Molecular Sciences*

MDPI

Article

Overexpression of *β-Ketoacyl CoA Synthase 2B.1* from *Chenopodium quinoa* promotes suberin monomers production and salt tolerance in *Arabidopsis thaliana*

Faheem Tariq[1,†], Shuangshuang Zhao[1,†], Naveed Ahmad[2], Pingping Wang[1], Qun Shao[1], Changle Ma[1*] and Xianpeng Yang[1*]

1 Shandong Provincial Key Laboratory of Plant Stress, College of Life Sciences, Shandong Normal University, Jinan 250014, China
2 Institute of Crop and Germplasm Resource (Institute of Biotechnology), Shandong Academy of Agricultural Sciences, Jinan 250100, China
* Correspondence: machangle@sdnu.edu.cn; yangxp2006@sdnu.edu.cn
† These authors contributed equally to this work.

Citation: Tariq, F.; Zhao, S.; Ahmad, N.; Wang, P; Shao, Q.; Ma, C.; Yang, X. Overexpression of *β-Ketoacyl CoA Synthase 2B.1* from *Chenopodium quinoa* promotes suberin monomers production and salt tolerance in Arabidopsis. *Int. J. Mol. Sci.* **2022**, *23*, 13204. https://doi.org/10.3390/ijms232113204

Academic Editors: Shaojun Dai and Chunzhao Zhao

Received: 10 September 2022
Accepted: 26 October 2022
Published: 30 October 2022

Publisher's Note: MDPI stays neutral with regard to jurisdictional claims in published maps and institutional affiliations.

Copyright: © 2022 by the authors. Licensee MDPI, Basel, Switzerland. This article is an open access article distributed under the terms and conditions of the Creative Commons Attribution (CC BY) license (https://creativecommons.org/licenses/by/4.0/).

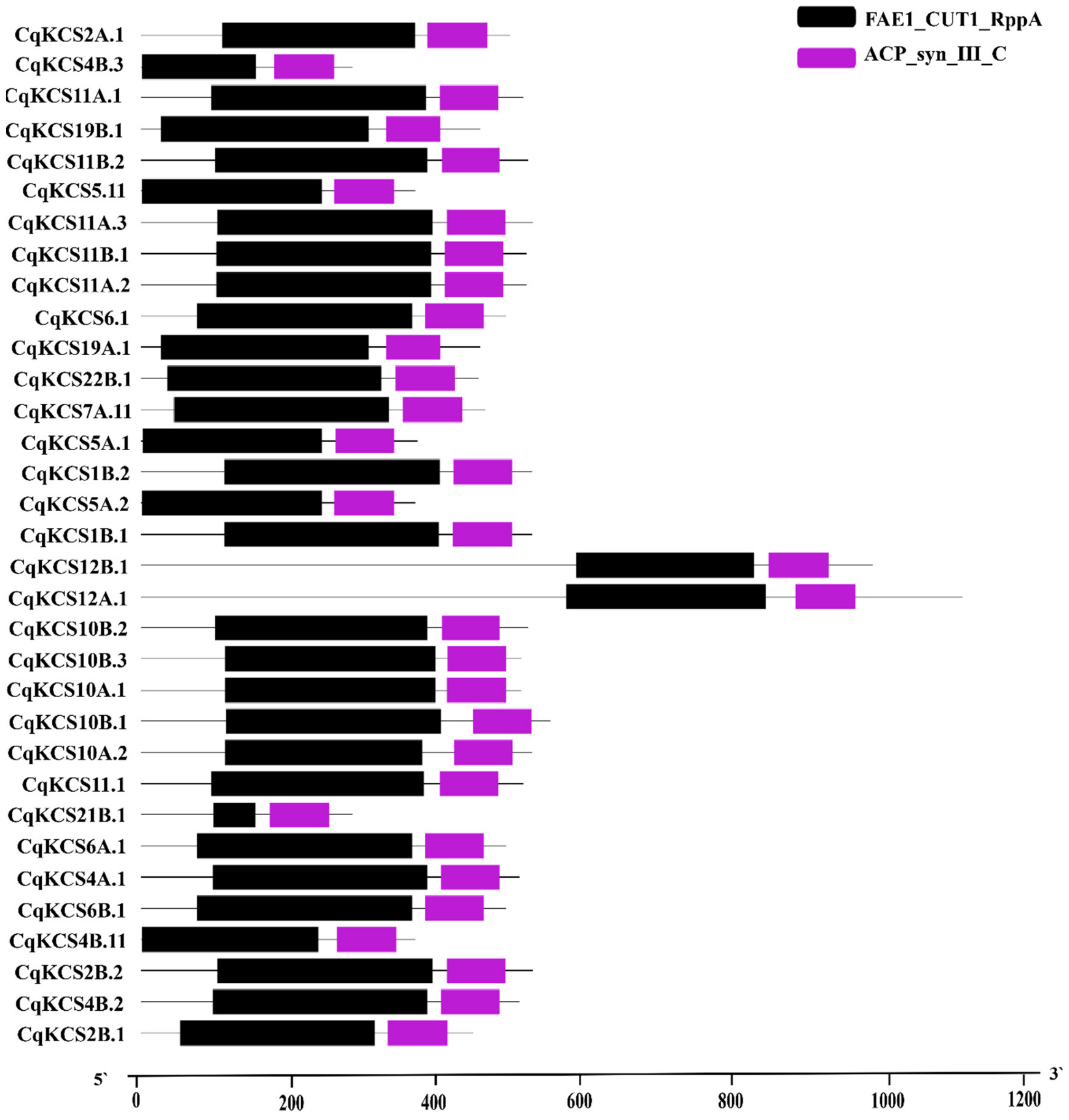

Supplementary Figure S1. The conserved domain of CqKCS from quinoa. The 33 CqKCS protein sequence contains two conserved domains i.e., FAE_CUT1_RppA (PF08392) and ACP_syn_III_C (PF08541).

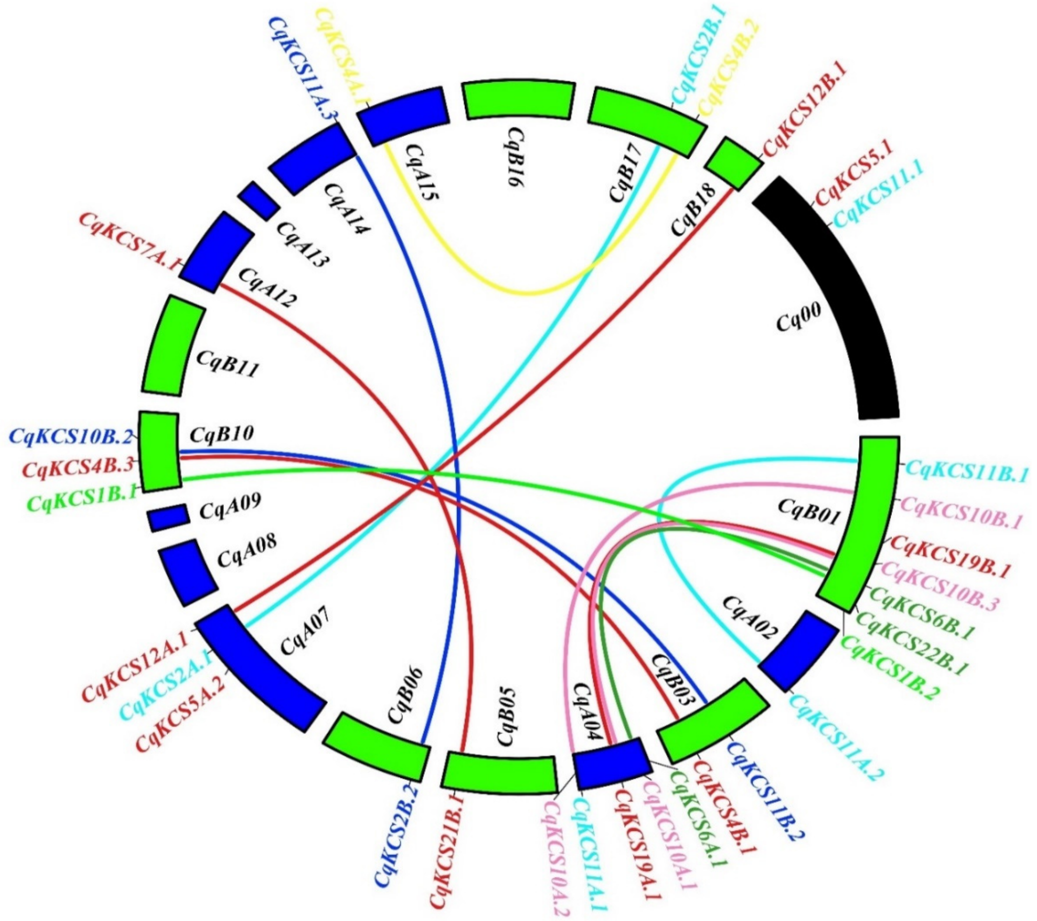

Supplementary Figure S2. Circos diagram of the CqKCS duplicated gene pairs in quinoa. The chromosome number is displayed inside the track, and the subgenome is indicated by the letters A and B, followed by the chromosome number. The joining lines represent CqKCS gene pairs that have been replicated between the A and B sub-genome. Outside of the chromosome, a gene's name is represented. The non-joined lines of KCS genes are not duplicated or non-syntenic.

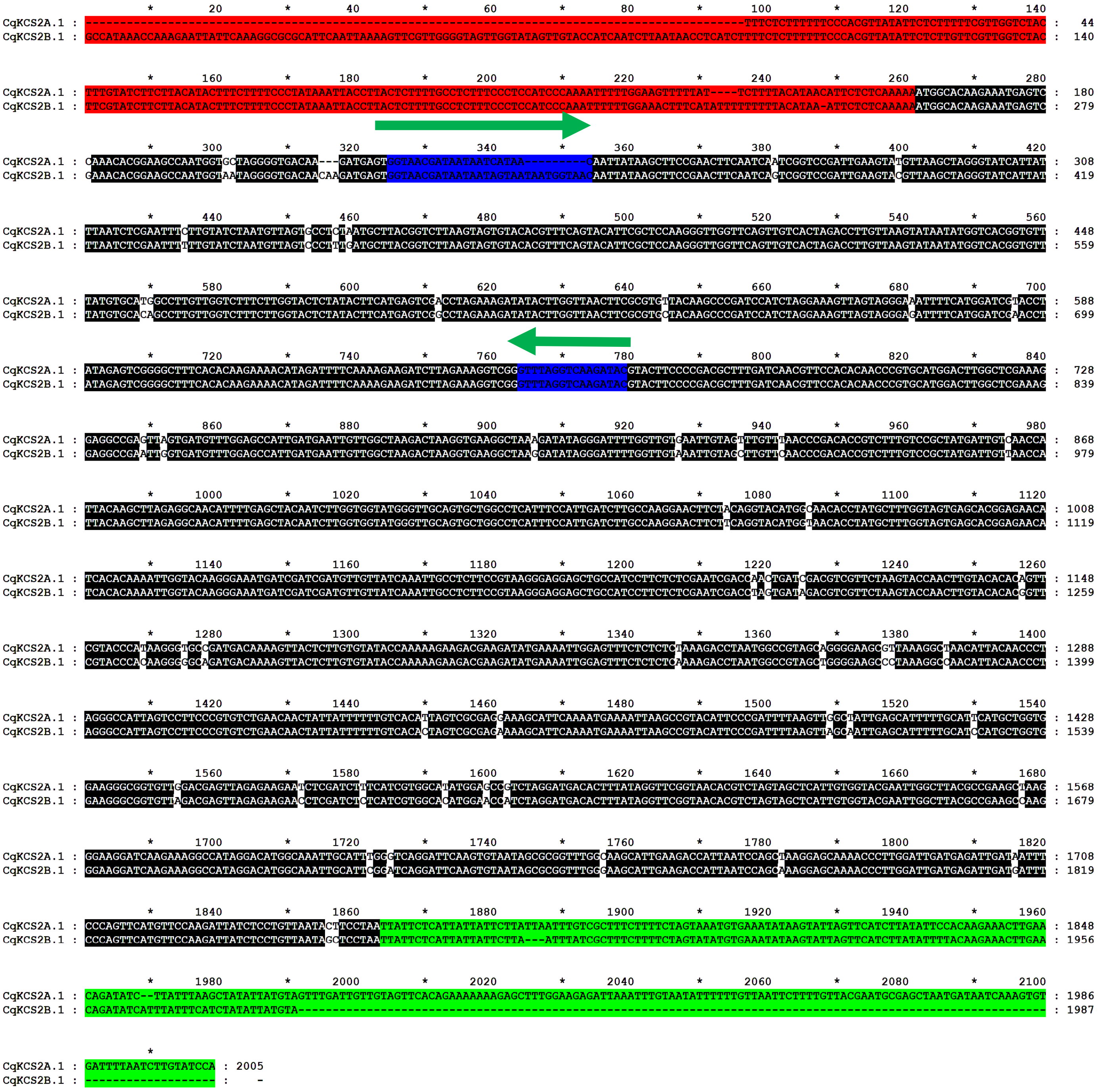

Supplementary Figure S3. The aligned sequence of CqKCS2A.1 and CqKCS2B.1 containing Semi RT-qPCR primers. Red color indicates 5 UTR, the green color indicates 3 UTR, the blue color indicates the primer attachment sequence. However, the forward primers used were different for CqKCS2A.1 as shown in supplementary material. Whereas, the reverse primer used was the same.

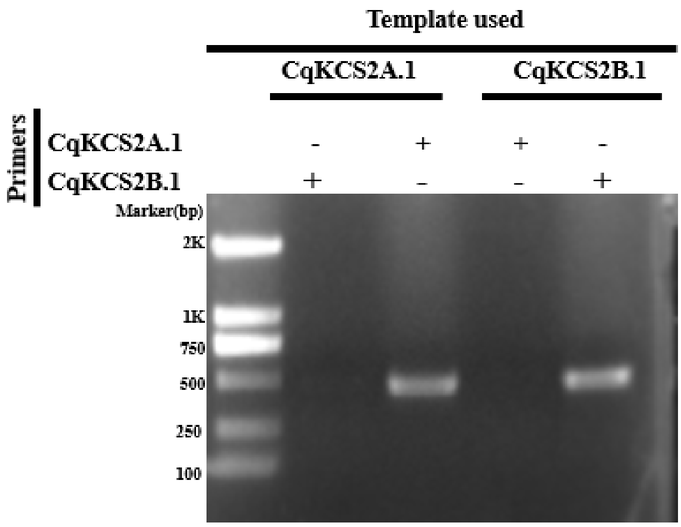

Supplementary Figure S4. CqKCS2A.1 and CqKCS2B.1 primer specificity in quinoa for semi-RT-qPCR.

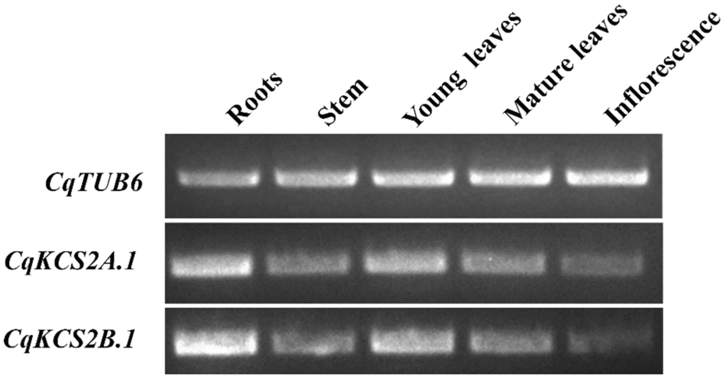

Supplementary Figure S5. The CqKCS2A.1 and CqKCS2B.1 expression analysis among different tissues of *C. quinoa*.

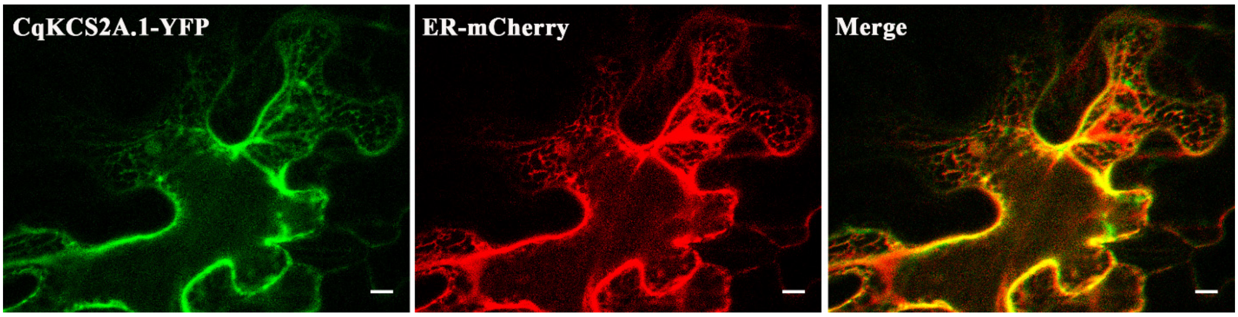

Supplementary Figure S6. *CqKCS2A.1* is localized to the Endoplasmic reticulum. Bar = 10 μm

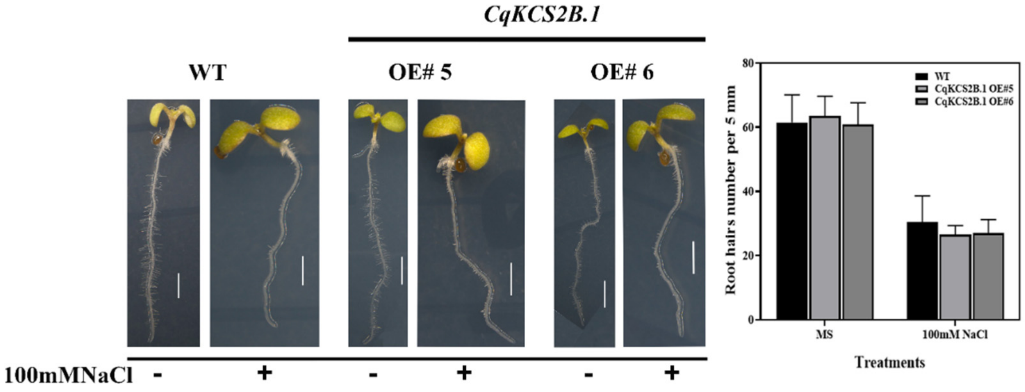

Supplementary Figure S7. Roots hair length and density of CqKCS2B.1 increases under salt stress. The statistical data significance was evaluated by ANOVA. Scale = 2mm.

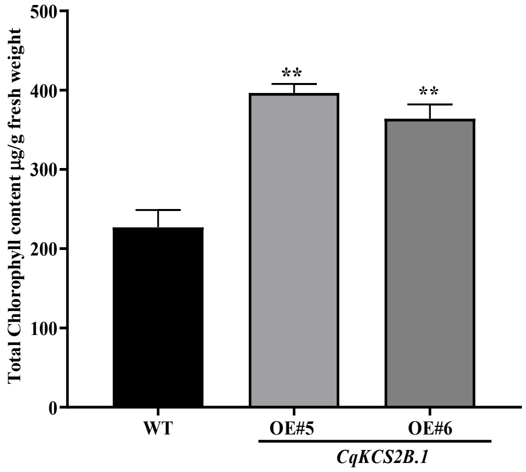

Supplementary Figure S8. Total chlorophyll analysis of CqKCS2B.1 transgenic lines transgenic. The statistical data significance was evaluated by ANOVA. Different * indicates the significant difference between different transgenic and WT at $P<0.05$. However, ** indicates $p<0.01$. scale=1cm.

S. No	Gene ID	Gene Name	Chromosome Name	Gene Start (bp)	Gene End (bp)	Gene Orientation	Protein Length (AA)	PI	Molecular weight (KDa)	Instability index	GRAVY
1	AUR62020751-RA	CqKCS10A.1	CqA04	18503386	18505576	F	511	8.95	57749.39	40.36	-0.17
2	AUR62033438-RA	CqKCS11B.1	CqB01	20017552	20019108	F	518	9.1	58113.63	42.33	-0.07
3	AUR62035979-RA	CqKCS19A.1	CqA04	22661716	22663083	F	455	8.57	52114.46	32.8	-0.15
4	AUR62024778-RA	CqKCS10B.1	CqB01	46501790	46498730	R	550	9.15	61975	43.59	-0.19
5	AUR62035224-RA	CqKCS5A.1	CqA14	7608832	7609947	R	371	8.89	41035.74	43.09	-0.05
6	AUR62034881-RA	CqKCS4B.1	CqB03	62068585	62069691	R	368	8.55	40479.47	34.64	-0.08
7	AUR62005216-RA	CqKCS21B.1	CqB05	72276953	72277969	F	284	9.14	32798.85	33.24	-0.205
8	AUR62016077-RA	CqKCS5A.2	CqA07	77110018	77108912	F	368	9.39	41113.12	43.67	-0.04
9	AUR62041842-RA	CqKCS11.1	Chr00	49761046	49759505	R	513	8.86	58046.41	42.64	-0.08
10	AUR62006329-RA	CqKCS2A.1	CqA07	89453807	89456293	R	496	9.31	56167.08	37.81	-0.19
11	AUR62041628-RA	CqKCS19B.1	CqB01	100574769	100576136	F	455	8.57	52109.44	34.25	-0.15
12	AUR62044250-RA	CqKCS6.1	Chr00	133275289	133277859	F	491	9.19	55558.22	37.32	-0.01
13	AUR62042172-RA	CqKCS11B.2	CqB03	35705846	35710413	R	520	9.36	58357.16	29.63	-0.03
14	AUR62000752-RA	CqKCS7A.1	CqA12	8670912	8672537	R	463	9.3	52064.73	32.96	0.038
15	AUR62016745-RA	CqKCS4B.2	CqB17	74376826	74378352	F	508	9.34	57278.72	33.93	-0.01
16	AUR62032347-RA	CqKCS11A.1	CqA04	51753194	51754735	R	513	8.86	57915.26	40.19	-0.06
17	AUR62032441-RA	CqKCS10A.2	CqA04	53017692	53020633	F	525	9.12	58950.43	43.23	-0.2
18	AUR62026627-RA	CqKCS4B.3	CqB10	23561607	23562461	R	284	9.11	31568.16	33.28	-0.185
19	AUR62026367-RA	CqKCS2B.1	CqB17	58497811	58500217	R	446	9.18	50287.45	37.66	-0.08

20	*AUR6201131 1-RA*	*CqKCS4A.1*	*CqA15*	*9345789*	*9347315*	*R*	*508*	*9.43*	*57359.81*	*34.47*	*-0.04*
21	*AUR6202266 4-RA*	*CqKCS1B.1*	*CqB10*	*5432330*	*5433907*	*F*	*525*	*9.19*	*59128.71*	*30.47*	*-0.09*
22	*AUR6201106 1-RA*	*CqKCS11A. 2*	*CqA02*	*5618308 2*	*5618463 8*	*F*	*518*	*9.02*	*58051.53*	*42.7*	*-0.06*
23	*AUR6200185 1-RA*	*CqKCS12A. 1*	*CqA07*	*1051719 87*	*1051853 27*	*F*	1105	8.61	126012.01	46.77	-0.368
24	*AUR6200898 4-RA*	*CqKCS6A.1*	*CqA04*	*6081797*	*6084367*	*F*	*491*	*9.19*	*55558.22*	*37.32*	*-0.01*
25	*AUR6202331 9-RA*	*CqKCS6B.1*	*CqB01*	*1144851 08*	*1144875 64*	*R*	*491*	*9.27*	*55525.22*	*36.28*	*-0.01*
26	*AUR6202331 4-RA*	*CqKCS22B. 1*	*CqB01*	*1145151 91*	*1145165 55*	*F*	*454*	*9.36*	*51266.04*	*45.91*	*-0.04*
27	*AUR6202128 6-RA*	*CqKCS5.1*	*Chr00*	*3655763 1*	*3655873 7*	*F*	368	9.46	40713.69	40.02	-0.035
28	*AUR6200455 1-RA*	*CqKCS1B.2*	*CqB01*	1202354 50	1202370 27	*F*	*525*	*9.19*	*59280.95*	*31.02*	*-0.09*
29	*AUR6200589 7-RA*	*CqKCS11A. 3*	*CqA14*	*5900965 2*	*5901267 4*	*F*	*527*	*9.09*	*59581.46*	*35.59*	*-0.08*
30	*AUR6202241 6-RA*	*CqKCS10B. 2*	*CqB10*	*2880196 4*	*2880683 5*	*R*	*520*	*9.18*	*58156.88*	*29.91*	*-0*
31	*AUR6200964 4-RA*	*CqKCS12B. 1*	*CqB18*	*2806779 0*	*2807407 4*	*F*	984	8.45	111885.4	45.64	-0.363
32	*AUR6202796 2-RA*	*CqKCS10B. 3*	*CqB01*	*1035277 13*	*1035298 57*	*R*	*511*	*8.94*	*57693.65*	*38.97*	*-0.13*
33	*AUR6203288 5-RA*	*CqKCS2B.2*	*CqB06*	*6748608*	*6751744*	*F*	*527*	*9.13*	*59614.66*	*32.1*	*-0.05*

Supplementary Table S1. Physiochemical properties of CqKCS proteins. Protein identification of all CqKCS from *C. quinoa* with their corresponding chemical and physical characteristics including gene id, protein length, molecular weight (MW; Da), isoelectric points, instability index and GRAVY. EPASY tool was used to collect the data http://web.expasy.org/protparam/.

**Vector construction primers**	
CqKCS2B.1-YFPF	ACATTTACAATTACGGATCCATGGCACAAGAAATGAGTCGAAACACGG
CqKCS2B.1-YFPR	TCGCCCTTGCCCATGGATCCGGAGCTATTAACAGGAGATAATCTTGGAAC
CqKCS2A.1-YFPF	ACATTTACAATTACGGATCCATGGCACAAGAAATGAGTC
CqKCS2A.1-YFPR	TCGCCCTTGCCCATGGATCCTTAGGAAGTATTAACAGGAGAT
**qRT-PCR**	
CqTub6. FP	GTTCAGGAGAGTAAGCGAGCAGTTC
CqTub6. RP	CTCTTCCTCATCGGCGGTAGCA
CqCMO. FP	TCCAAAGCAAGCGAAGAACAGTCA
CqCMO. RP	TTCAGCACAACTACCAAGCCATTCA
CqKCS2. FP	ACCGTCTTTGTCCGCTATGATTGTT
CqKCS2. RP	TGATGTTCTCCGTGCTCACTACC
**Semi RT PCR**	
CqKCS2B.1 RT-F	GGTAACGATAATAATAGTAATAATGG
CqKCS2B.1 RT-R	GTACGTATCTTGACCTAAAC
CqKCS2A.1 RT-F	GTGGTAACGATAATAATCATAAC
CqKCS2A.1 RT-R	GTACGTATCTTGACCTAAAC
CqTUB6 FP	TGAGAACGCAGATGAGTGTATG
CqTUB6 RP	GAAACGAAGACAGCAAGTGACA
CqCMO FP	TTGTTGCTAGAGTTGAGCGT
CqCMO RP	AGGGGTGCAATGGTGTTTAT
**Lateral root associated genes Primers**	
PUCHI qRT FP	ACGGCTCGTTATCTTCTTCACT
PUCHI qRT RP	TGGACTTATTATGTTCTTCGCTTG
GH3.3-qRT FP	ACAATTCCGCTCCACAGTTC
GH3.3-qRT RP	ACGAGTTCCTTGCTCTCCAA
GH3.5qRT FP	GTCTTCGAGGACTGCTGCTT
GH3.5-qRT RP	ATGTCCCTGGCTCAACAATC
AUX1 qRT FP	GAGGTCACGCGGTTACTGTT
AUX1 qRT RP	ATAAGAGAGAAAGCGTTGGAGTGG
LBD29-qRT FP	GCTAGGCTTCAAGATCCCATC
LBD29-qRT RP	TGTGCTGCTTGTTGCTTTAGA
GATA23-qRT FP	TAAGACCACCAAGACACCAATG
GATA23-qRT RP	ACAAAGCAGCTTGTTCTTCCTC
LBD18-qRT FP	TCATCGAAGGTCCGATGCTGTC
LBD18-qRT RP	GCCTGTAGATTCACCACCTGTTGC
Actin 2 FP	CTTGCACCAAGCAGCATGAA
Actin 2 RP	CCGATCCAGACACTGTACTTCCTT
LBD16 qRT FP	TACAACGGCGGGGACAGGT

LBD16 qRT RP	GCTGCGAATCTTGCTGCTCC

Supplementary Table S2. List of Primers used in this study
